# Impact of olfactory disorders on personal safety and well-being: a cross-sectional observational study

**DOI:** 10.1007/s00405-024-08529-9

**Published:** 2024-02-23

**Authors:** Liam Lee, Louis Luke, Duncan Boak, Carl Philpott

**Affiliations:** 1 University of East Anglia Medical School, University of East Anglia, Norwich, UK; 2 Ear, Nose and Throat (ENT) Department, James Paget University Hospital, James Paget University Hospitals NHS Foundation Trust, Great Yarmouth, UK; 3 Fifth Sense, Unit 2, Franklins House, Wesley Lane, Bicester, OX26 6JU UK

**Keywords:** Olfactory dysfunction, Anosmia, Hyposmia, Safety, Observational

## Abstract

**Objectives:**

Investigate safety perceptions, quantify hazardous events, and analyse their manifestations in individuals with olfactory dysfunction through an online cross-sectional survey.

**Methods:**

An online survey, available from 25th February to 28th September 2022, captured data on demographics, olfactory disorder causes, safety concerns, and experienced hazardous events. Distributed via Fifth Sense channels, it targeted individuals with self-claimed olfactory dysfunction.

**Results:**

Of 432 responses, the majority were female (79.6%), aged 41–70, with 20.6% non-UK residents from 21 countries. Leading causes of dysfunction were Covid-19 (22%), idiopathic (20.8%), and congenital (14.4%). Safety concerns were high (85.9%), with gas, smoke, and food as major worries. Over 5 years, 32.2% faced ≥ 1 food incident, 14.8% ≥ 1 gas incident, 34.5% ≥ 1 gas scare, and 18.5% ≥ 1 work incident. Preventative measures were taken by 60.2% at home. Key limitations of this study were self-reported data and sampling bias of charity members.

**Conclusion:**

This study highlights the significant impact of smell loss on personal safety and emotional well-being. There is an unmet need in mitigating safety concerns/events for individuals with olfactory dysfunction. We suggest collaborate strategies such as educating the public sector and high-risk sectors (e.g. gas companies), and introducing safety ‘scratch and sniff’ cards as a screening method. Regular assessment of an individual’s olfactory ability, similar to routine assessments for other sensory systems (sight, hearing) may allow proactive identification of at-risk people and corrective measures to take place.

**Supplementary Information:**

The online version contains supplementary material available at 10.1007/s00405-024-08529-9.

## Introduction

### Background and rationale

Loss of smell is a common but debilitating condition. It is estimated that the prevalence of either complete loss of smell (anosmia) or incomplete loss of smell (hyposmia) is approximately 2.7 to 24.5% in population-based studies based on objective olfactory assessment [1]. Most common acquired causes of olfactory dysfunction are predominantly sinonasal conditions and viral infections [2]. Loss of smell disproportionally affects older people and the inability to perceive odours can lead to significant safety hazards [3]. For instance, individuals with impaired olfaction are at a heightened risk of not detecting smoke, gas leaks, or spoiled food, which are critical for avoiding danger and maintaining personal hygiene [4].

However, people with olfactory dysfunction remain understudied and under-represented compared to other ear, nose and throat conditions. While the prevalence of safety risks in individuals with olfactory dysfunction has been quantitatively demonstrated in prior studies, there is a noticeable gap in the literature regarding the incidence of hazardous events and large-scale surveys focussing on the incidence and nature of safety scares are scarce [4–8]. One study that investigated the incidence of safety hazards reported 30–35% experienced at least one hazardous event the past 10 years, but this has remained stagnant across 3 decades [9]. In addition, only few studies captured the qualitative aspect and the nuances of how these risks manifest, which are crucial in tackling mitigating strategies [6, 10]. Consequently, there has been limited progress in mitigating these safety risks. Previous survey study, such as Philpott and Boak, highlighted the safety aspects of smell impairment, suggesting the safety issue remains a concern [11]. With Covid-19 and an ageing population contributing to the increased prevalence of olfactory dysfunction, it is important to revisit this problem through large-scale survey studies and tackle the challenges through appropriate support and protection [12].

### Objectives

We sought to investigate the perception of safety reported by individuals with loss of smell. We quantified safety concerns, estimated incidence of safety hazards, and how safety scares/incidents manifest. The findings from this survey will be significant in identifying unmet needs in providing safety support for individuals with loss of smell.

## Materials and methods

### Study design

This study is an online cross-sectional survey exploring the personal safety and emotional well-being of those affected with olfactory dysfunction. The survey was created on SurveyMonkey, designed to capture both quantitative and qualitative data. Data included in analyses were responses from 25th February 2022 to 28th September 2022. The survey remains open for continuous data collection.

As the survey was anonymous and considered to be service evaluation, there was no ethical approval sought in line with the Health Regulation Authority guidance: http://www.hra-decisiontools.org.uk/research/docs/DefiningResearchTable_Oct2017–1.pdf.

### Setting

Data were collected through the UK charity Fifth Sense which supports people affected by smell and taste disorders. The survey was shared via Fifth Sense email newsletter and social media channels and open to individuals worldwide. The participants could access the survey online free of charge.

### Participants

Anyone with a formal medical diagnosis or those who self-identified as having a problem with their sense of smell were eligible to participate in the survey.

### Data sources and variables

The survey had a total of 18 questions. We collected demographics (gender, age, profession, and region of residence), details about olfactory dysfunction (cause and duration), degree of safety concern, frequency hazardous events and its effect on day-to-day living. Survey comprised of a mix of yes/no, multiple-choice, rating scale questions, and free text. Full survey is available here: https://www.surveymonkey.co.uk/r/2BR3XTC.

### Variables and measurements

Our survey focussed on key variables to assess the impact of olfactory dysfunction on safety. Cause of smell loss: determined via a multiple-choice question covering common causes such as sinonasal conditions, viral infections and head trauma. Safety concerns: participants rated their concern about missing critical odours (like smoke or gas leaks) on a Likert scale, providing a quantifiable measure of perceived risk. Hazardous events: we asked participants to report the frequency (minimum 0 to maximum 5+) in the last 5 years due to smell loss, and optional free-text to explain the situation.

### Bias

As the survey was distributed online via a UK-based charity, there is a selection bias as participants are likely members seeking support, as well as selection bias to those who have internet access and are more likely to be UK residents. In addition, individuals with more significant olfactory dysfunction may be more likely to respond. This may subsequently overestimate the safety impact of olfactory dysfunction. There is a risk of response bias as participants tend to agree or provide positive answers in, e.g. rating scales and yes/no questions. We aimed to mitigate this issue by incorporating free-text boxes to allow participants to be more nuanced. Finally, we recognise the limitations inherent in self-reported data, including tendencies for inaccurate or biased information.

### Study size and statistical methods

No minimum sample size was required for the study as no statistical analyses were performed. Only descriptive analyses were performed given the nature of the data. All figures were created on R using ggplot2 package [13].

## Results

### Participants

**Fig. 1:**
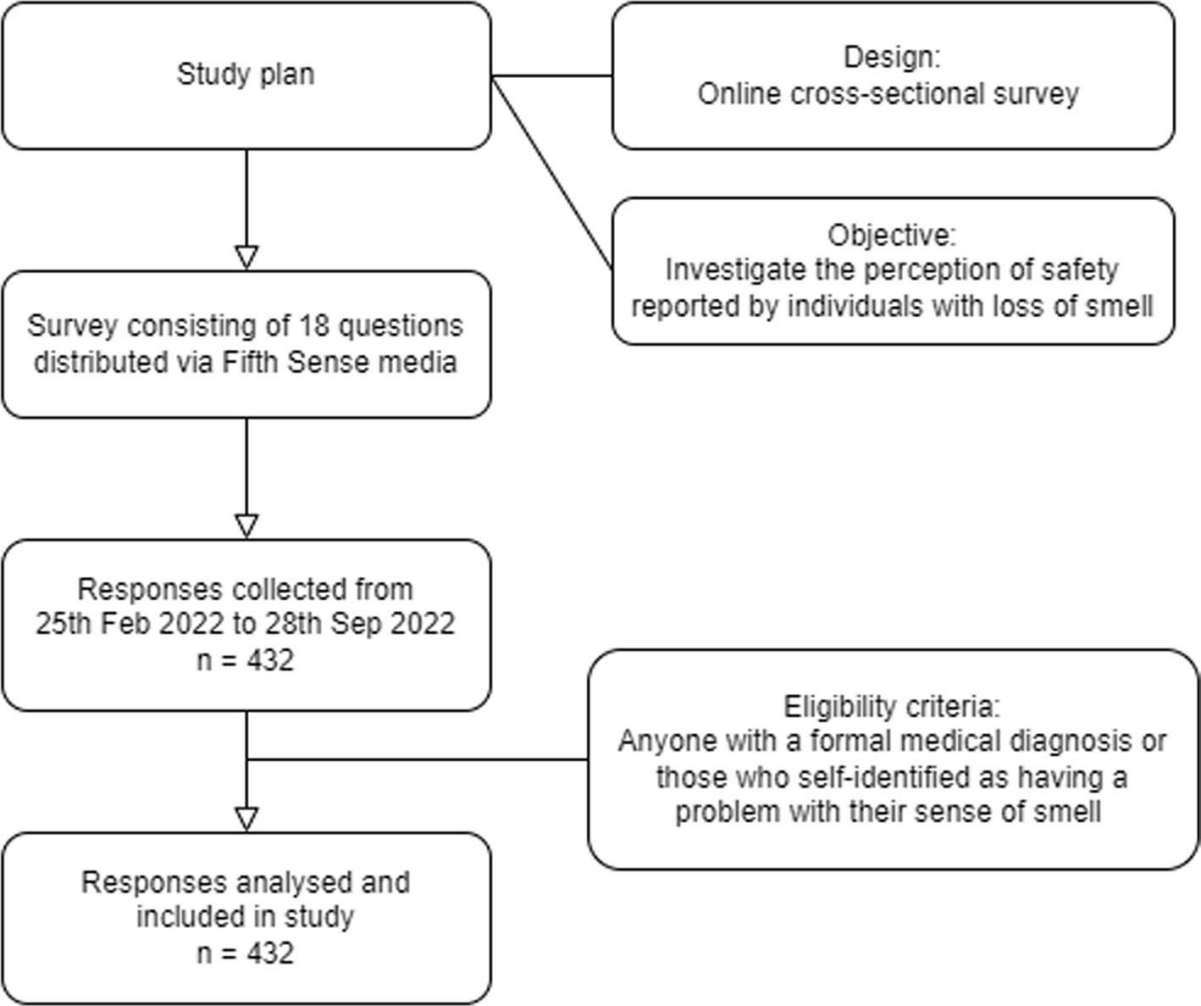
Flowchart of study process

The survey gathered responses from 432 participants. Answers from all 432 participants were analysed in the study. Flowchart of study process seen in Fig. 1.

### Descriptive data

Patient demographics are summarised in Table 1. Out of the participants, most participants were female (79.6%), followed by male (18.5%). The most common age groups were between 41 and 55 and 56 and 70. A quarter stated they were retired (26.9%). Majority of respondents were from England, but a significant portion (16.7%) were from across the world with 48 out of 72 participants abroad from United States.

**Table 1:** Participant demographics

Variable	Frequency (%)
Gender	
Female	344 (79.6%)
Male	80 (18.5%)
Gender neutral	4 (< 1%)
Transgender	1 (< 1%)
Prefer not to say	3 (< 1%)
Age	
Under 18	12 (2.78%)
18–25	13 (3.01%)
26–40	40 (9.26%)
41–55	141 (32.6%)
56–70	161 (37.3%)
70 +	65 (15.0%)
Profession (top 3 answers)	
Retired	116 (26.9%)
Teacher	19 (4.4%)
Administrator	13 (3.1%)
Region	
England	290 (67.1%)
Scotland	32 (7.41%)
Wales	14 (3.24%)
Northern Ireland	6 (1.39%)
Channel Islands	1 (0.23%)
Abroad	72 (16.7%)
NA	17 (3.9%)

### Main results

#### Causes of olfactory dysfunction

Covid-19 infection was reported by 95 people (22%) as cause for their olfactory dysfunction, closely followed by idiopathic reported by 90 people (20.8%). Remaining causes in descending order were congenital (62, 14.4%), post-traumatic head injury (61, 14.1%), viral infection other than Covid-19 (47, 10.9%), sinonasal disorder (46, 10.6%), and other causes (36, 8.3%). Within other causes, a myriad of aetiologies was mentioned including intracranial lesions (e.g. meningioma and chondrosarcoma), iatrogenic (secondary to brain/nasal operation), facial trauma and large doses of analgesia.

#### Safety concerns

**Fig. 2:**
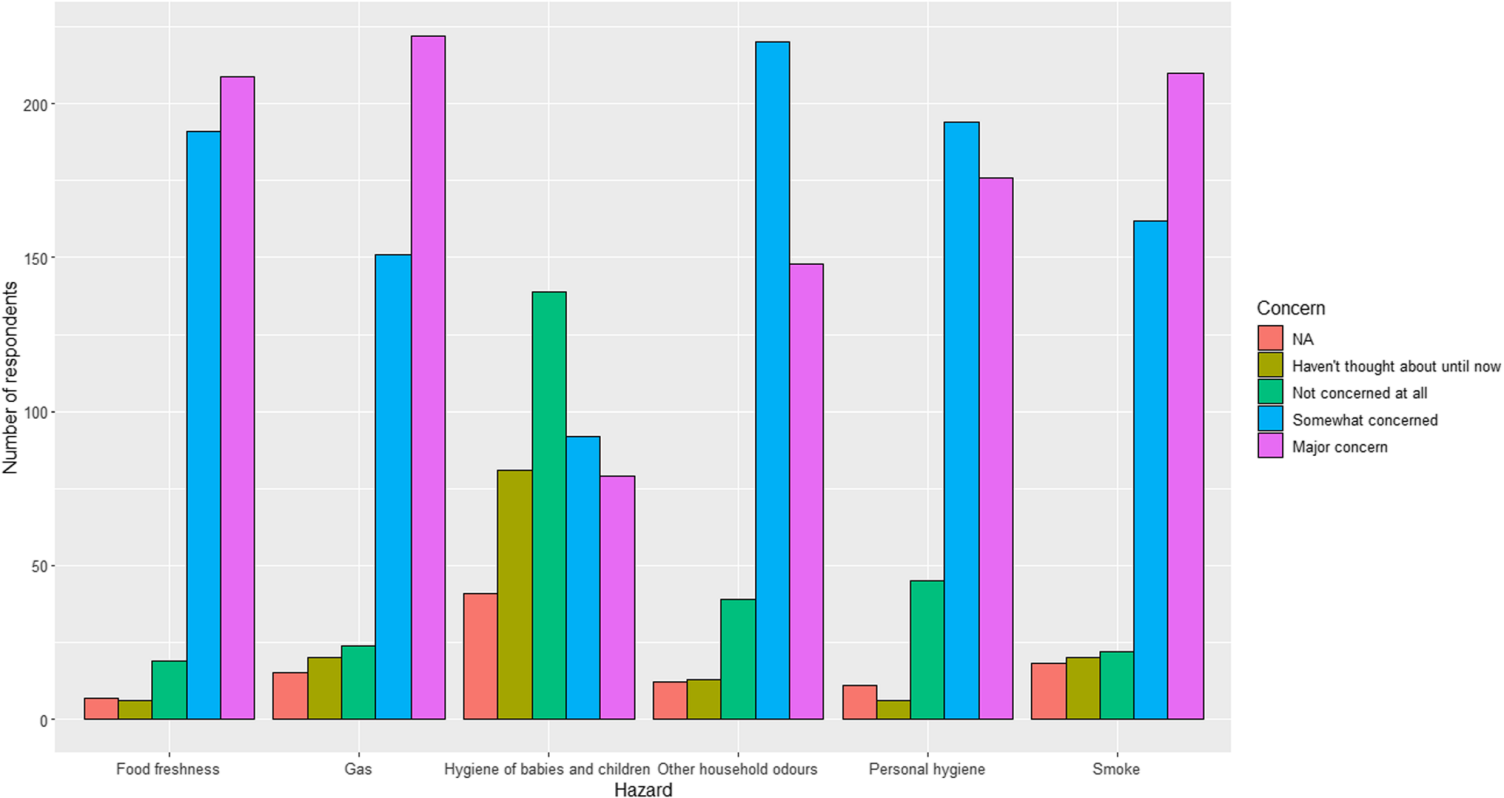
Degree of safety concerns for gas, smoke, food freshness, personal hygiene, hygiene of babies and children, and other household odours (e.g. waste bins or pets)

When asked about safety, there was an overwhelming response that stated they were worried about safety with 371 (85.9%) responding yes, 31 responding no (7.2%), and 30 who have not thought about it until now (6.9%). Gas, food freshness and smoke were safety categories where ‘major concern’ was the most popular answer (Fig. 2). Other household odours and personal hygiene, the most popular answer was ‘somewhat concerned’. For hygiene of babies and children, the most popular answer was ‘haven’t thought about until now’. A separate figure including only UK participants shared same trend of answers (Supplementary Material, Fig. 1).

Other worries written by the respondents outside the safety categories given were burning food when cooking, faulty exhausts in cars, perfume, and missing out on the smell of others.

#### Quantification and examples of hazardous events past 5 years

**Fig. 3:**
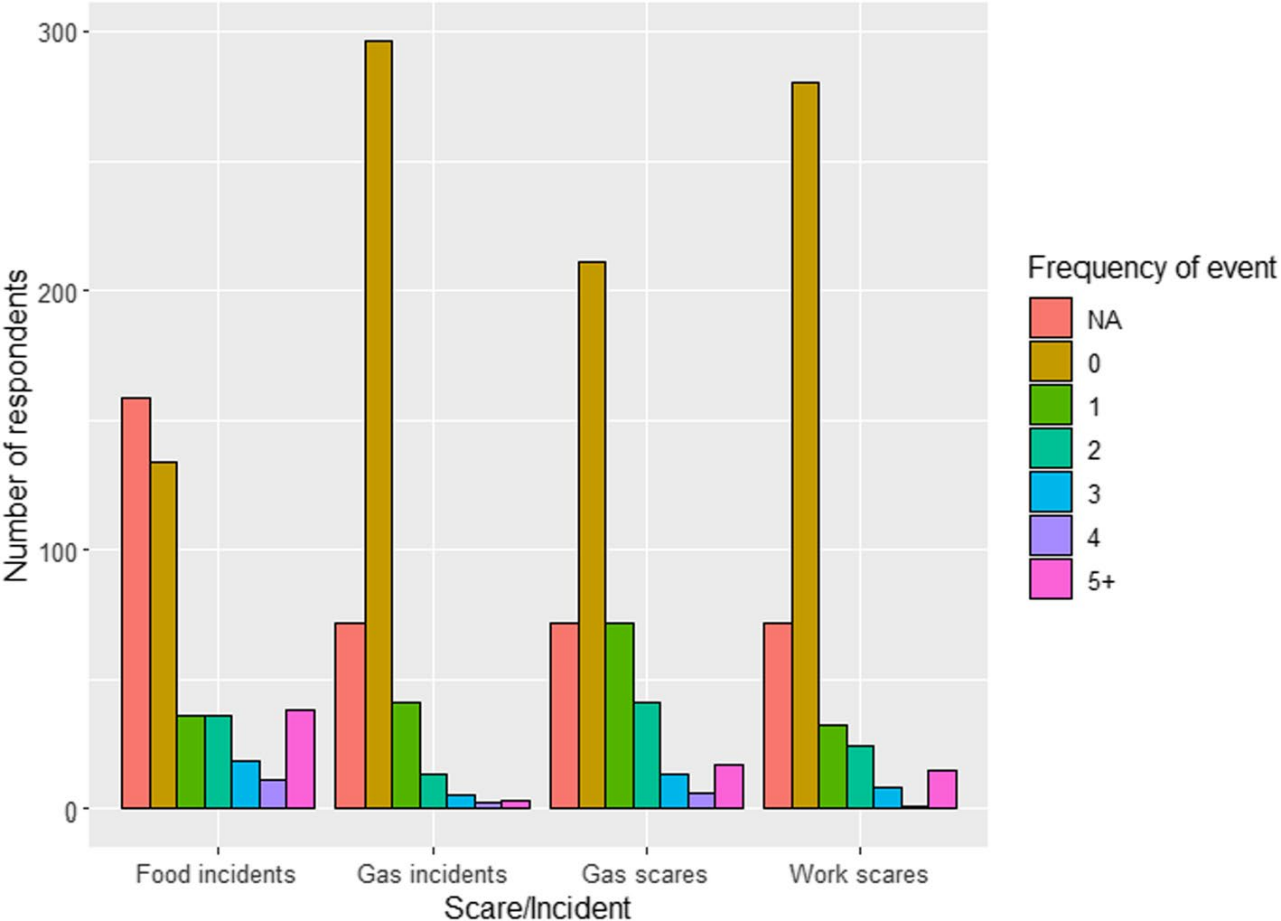
Scares and incidents in the last 5 years. We defined a ‘scare’ as a situation in which the individual was unable to smell but has not led to any harm, such as an appliance (e.g. cooker or hob) not being properly turned off. An ‘incident’ refers to a situation which the individual was at risk of serious harm or has led to harm, such as gas leak caused by anything other than an appliance, gas explosion, or ingesting faulty food

In our investigation of hazardous events experienced in the last 5 years, we discovered varied frequencies among different types of incidents (Fig. 3, Table 2). While most respondents reported not experiencing any adverse events, it is noteworthy that among those without any gas-related incidents, a common reason cited was the deliberate avoidance of living in environments with gas installations due to fear and anxiety of potential accidents.

**Table 2:** Scares and incidents in the last 5 years' numbers and percentage

Group	Food incidents (%)	Gas incidents (%)	Gas scares (%)	Work scares (%)
NA	159 (36.8%)	72 (16.7%)	72 (16.7%)	72 (16.7%)
0	134 (31.0%)	296 (68.5%)	211 (48.8%)	280 (64.8%)
1	36 (8.3%)	41 (9.5%)	72 (16.7%)	32 (7.4%)
2	36 (8.3%)	13 (3.0%)	41 (9.5%)	24 (5.6%)
3	18 (4.2%)	5 (1.2%)	13 (3.0%)	8 (1.9%)
4	11 (2.5%)	2 (0.5%)	6 (1.4%)	1 (0.2%)
5 +	38 (8.8%)	3 (0.7%)	17 (3.9%)	15 (3.5%)

In terms of the most commonly experienced hazardous events, our data revealed that food-related incidents were most frequent, with 32.2% of participants reporting at least one such occurrence. This was followed by gas scares (34.5%), work-related scares (18.5%), and gas incidents (14.8%). When the hazard types were combined, around 45% experienced at least one hazardous event of any kind. The trend was consistent even when focussing solely on UK participants: 29.7% for food incidents, 34.8% for gas scares, 15.3% for work scares, and 14.7% for gas incidents (Supplementary Material, Fig. 2).

The responses frequently contained words such as “worry,” “concern,” “anxiety,” and “paranoid.” For example, one respondent described a habitual paranoia about gas safety, leading to repetitive checking and persistent anxiety. Despite such precautionary efforts, the quantified percentages indicate that a significant portion of the participants have faced at least one type of hazardous event related to their olfactory dysfunction. Examples of hazardous events are provided in Table 3.

**Table 3:** Examples of events experienced by individuals suffering from olfactory dysfunction

Type	Examples
Gas safety scare	“I have left gas hob on a few occasions partner had to raise awareness when in from work I thought I had turned it off” “A design flaw in my hob meant the gas could be accidentally switched on by a small amount. Luckily my partner picked up on this as I had no idea each time. We have since switched to an induction hob, and only have gas for the central heating boiler.” “The gas oven we have has an eye level grill. It lit and I had turned around to prepare the next thing for cooking. My Mum came into the kitchen and smelt gas so checked the grill and the flame had gone out but the gas was still on. We had to open all the windows and doors at the bottom of our house and wait for the gas to dissipate. I no longer cook with the gas oven unless someone else is there to supervise.” “Not aware the oven flame had gone out, but the gas was still on. The same has happened on the hob. I also live on my own, so would feel a lot happier if I had something which would detect any gas leaks.”
Gas safety incident	“I had a gas leak from raw gas straight from pipes under my bed. Ended up in hospital on oxygen and my street was evacuated until fire and gas officers made it safe.” “My gas scare safety was more than 5 years ago, but was very serious. Building works next door had caused earth movements, cracking the gas main. A neighbour knocked on the door wondering why I hadn’t reported it, not knowing me as I was new to the area, and she was trying to be respectful. The gas company arrived within 15 min, and shut the gas to the entire street, it was apparently a very big leak.” “I have parosmia as part of Long Covid and thought I smelt damp in my kitchen. My carer, who also has Long Covid, could smell damp too. But we couldn’t pin down the source. 4 days later, my other carer came back from holiday and told me the smell was gas, not damp! She called the gas board who visited and confirmed I had a gas leak in my kitchen.”
Food safety incident	“Almost ate chicken that smelled bad, luckily son said it was very smelly otherwise it was going into a crockpot with sauce.” “Regular scares (5 +) for off off milk. I can only tell when the milk curdles in my tea. I will not eat food past its sell by date and if in doubt I throw away. Designated nose is important.” “I don’t take any risks. I throw food out before it has a chance to go off. As a result, I buy very little food and I never cook.” “When I could not taste it was at least a couple of times per week for the 6 months, now, meat onion and garlic are so overwhelmingly terrible it hides other smells and taste. I had salad and cheese several times that gave me problems and a few times with soups and vegetable stews. Now I throw things out on the third day. A terrible waste but I can’t tell if it is ok”
Safety scares in the workplace	“A pan was on fire and as I was not looking towards the kitchen I could not smell the smoke.” “Couple of times very strong chemicals were used at work in toilets and I was totally unaware until boss told me to get out. I did start to feel lightheaded.” “I used to visit customer properties and was unable to smell gas leak and other substances that I needed to be aware of.” “Very many [scares/incidents] including nearly burning down the staff room when a potato caught fire in a dodgy microwave. Gas fire over teachers bench in lab.”

#### Mitigations

Approximately, 60% of participants implemented home measures to mitigate the risks associated with olfactory dysfunction. These measures included installing gas detectors, exercising extra caution (such as discarding food past its best before date or avoiding gas usage altogether), and depending on someone else's sense of smell for assistance. On the employment front, about 43% reported that their employers had taken steps to ensure safety, including the installation of smoke/gas detectors, requiring work to be done in pairs, and providing additional protection against Covid-19. It is important to note, however, that around a quarter of the respondents were retired and hence considered this question inapplicable to their current situation.

## Discussion

### Key results

This international survey highlights the impact of olfactory disorders on personal safety. Post-viral olfactory loss including Covid-19 accounted for 32.9% of respondents, highlighting the increasing prevalence of olfactory dysfunction since the start of the pandemic. Irrespective of the cause, 85.9% were concerned about safety. Despite precautionary measures taken by majority of respondents, the percentages of at least one hazardous experience were 32.2%, 14.8%, 34.5%, and 18.5% in the order of food incident, gas incident, gas scare, and work scare. There was a recurrent use of words that suggested an underlying anxiety that led to precautionary measures in fear of having an accident.

### Strengths and limitations

It is essential to highlight the strengths of this cross-sectional survey. First, many respondents with a range of olfactory disorders participated in this survey shedding light on the impact of olfactory disorders on personal health and safety. Second, this survey quantified the occurrence of safety scares/incidents in the preceding 5 years allowing qualitative data to be collected detailing such events. Lastly, data were collected anonymously allowing respondents to give honest accounts of their experiences.

However, there are limitations with this study. First, all responses were self-reported, including the aetiology of olfactory dysfunction which could not be clinically verified. It is hard to comment on whether the aetiology is subjective from the participant or an objective diagnosis from a clinician. In fact, many normal smelling individuals claim themselves to be severely affected, and subjective assessment of olfactory function is known to be unreliable [14]. Future studies would benefit from formally diagnosed study sample. Second, there is a paucity of detailed demographic and medical information including socioeconomic status, ethnicity, co-morbidities, management options used which may affect the interpretation and generalizability of our results. Particularly pertaining to demographic information, there is evidence that olfactory dysfunction is more prevalent in lower socioeconomic status and ethnic minorities, which may subsequently pose an increased risk to safety hazards [15]. Lastly, reporting and recall bias is an issue in this study. All respondents were Fifth Sense members and, thus leading to bias in results. This specific demographic might have heightened awareness or concerns about olfactory dysfunction, potentially leading to reporting bias where participants overemphasise their experiences. In addition, recall bias could have influenced the accuracy of their memories, especially for events that occurred in the distant past. Nevertheless, it is important to note that our study group consists of individuals who are likely to seek medical help for their olfactory problems. This means that while our findings might not apply to everyone, they do provide insight into the experiences of those who actively seek clinical intervention for olfactory dysfunction.

### Interpretation

Post-viral olfactory loss, including Covid-19, was the most common aetiology of olfactory loss within our respondents. However, it is important to note that the literature presents varying reports on the prevalence of etiologies for olfactory loss. This variation can be attributed to differences in study design, geographical location, population demographics, and the definition and measurement of olfactory dysfunction used in each study. Nordin and Bramerson’s review showed that post-viral olfactory loss and sinonasal disorders are more common aetiologies for olfactory disorders than post-traumatic head injury and congenital olfactory loss [16]. In another survey conducted in ENT departments in German-speaking countries, the majority were secondary to sinonasal disorders (67%), then post-infective (14%), idiopathic (8%) and traumatic (6%), which varies compared to our results [17]. One explanation for the large proportion of post-viral olfactory loss in our study is due to the Covid-19 pandemic, as olfactory loss is a cardinal symptom that can persist for months after the acute infection and manifest as parosmia/phantosmia [18, 19]. Another possible explanation for the difference in the prevalence of olfactory disorders may be inherent to our study design. For instance, individuals with conditions that can be managed with medications or surgery (e.g. sinonasal disorders) may seek less support and are therefore less likely to come across the survey distributed by Fifth Sense. Congenital, post-traumatic, and post-viral olfactory loss have limited management options available, and participants are more likely to seek support and advice from charities.

Another observation from our survey is the heightened concern for food freshness, gas and smoke hazards compared to others, such as hygiene issues. This prioritisation likely reflects the immediate and potentially life-threatening nature of gas leaks or fires versus the more manageable risks associated with poor hygiene. While food freshness-related events are less immediately dangerous than gas-related scares, these food incidents are a constant concern due to their frequent occurrence. The frequency of food-related incidents, the most common of the hazardous events reported, underscores the ongoing challenge individuals with olfactory dysfunction face in ensuring food safety. Strikingly, our data suggested that 45% experienced at least hazardous event of any kind. In comparison with Pence et al. (2014), which found that 39.2% of clinically anosmic patients reported experiencing at least one hazardous event, as opposed to 18% among normosmic patients, our study presents a nuanced picture [9]. While direct comparisons are challenging due to differences in the incidence timeframe and potential sample biases in our study, the numbers we observed remain substantial. This potential increase in hazardous events could partly be attributed to heightened awareness and self-reporting of olfactory impairments in the general public, particularly in the wake of the Covid-19 pandemic. Supporting this notion, a safety questionnaire administered to individuals 6 months post-Covid-19, who suffered from olfactory dysfunction, revealed that as many as 57% had experienced at least one hazardous event [20]. This suggests a growing recognition and concern regarding the safety implications of olfactory loss in the current context.

An important finding in our survey is the impact of olfactory disturbance on a person’s quality of life, not just limited to physical safety and hygiene, but also their emotional well-being as a consequence of living in fear. While a majority of respondents had not experienced any hazardous events in the preceding 5 years, this is likely due to cautious day-to-day living to prevent a hazardous event from happening. Studies that performed thematic analyses identified olfactory disturbance to impact different aspects of life, including the detection of hazards, the feeling of social isolation, negative emotions including depression, and physical health [4, 10, 11, 21, 22]. Our findings echo the results of Keller and Malaspina’s online survey of 1000 patients with olfactory dysfunction, where 72% were concerned about hazard avoidance and the lack of food enjoyment [10]. Miwa et al. highlighted that 75% of participants were concerned about spoiled food and 61% were concerned about the failure to detect fire, gas or smoke [7]. These themes were concordant with other surveys’ results [8, 23]. In our survey, a recurring method to mitigate adverse events was having another person present in the house or workplace to help alert the respondent of danger. However, this mitigation is of no help to people who live alone. According to Office of National Statistics data, around 1 in 4 people over the age of 65 in the UK live alone.

### Generalisability

The safety implications are relevant for anyone with olfactory dysfunction, and protection for this population group is crucial. In the UK, a new legislation introduced in October 2022 made smoke and carbon monoxide detectors mandatory in both socially and private rented properties [24]. To the contrary, natural gas detectors is not commonplace and has no legal requirement for them to be installed. The cost of gas detectors can range anywhere from 20 to 5000 GBP depending on the specifications. There is a need for greater recognition of these safety risks, and education about the possible solutions, including making standardised, low-cost gas detectors widely available. In addition, the introduction of routine screening tests to identify individuals at higher risk due to olfactory dysfunction is vital. These tests could be integrated into regular health check-ups, particularly for populations known to be at greater risk, such as the elderly or those with a history of viral infections, and sinonasal disorder. Implementing these strategies would not only raise awareness but also significantly enhance the safety and well-being of individuals suffering from olfactory loss.

## Conclusion

We have demonstrated the safety concerns among individuals with olfactory dysfunction, incidence of hazardous events over the last 5 years, and highlighted the impact on mental well-being. There is a need for collaborative strategies from the government, healthcare sector and high-risk sectors such as gas companies to address this issue. Key areas of discussion would be the safety risks faced by individuals with olfactory dysfunction, cost-effective natural gas detectors to be made widely available along with simple tools such as scratch and sniff cards as a screening method to identify and protect vulnerable individuals susceptible to safety hazards. Routine assessment of olfactory ability in public health settings, particularly for older people, will also play a key role in identifying ‘at risk’ individuals.

### Supplementary Information

Below is the link to the electronic supplementary material.

Supplementary file1 (DOCX 1366 KB)

## Data Availability

This study is based on data collected from an anonymous survey. The survey did not include any personally identifiable information, and participants did not specifically consent to their data being shared beyond the research team.
