# Supplementary material for: Impact of olfactory disorders on personal safety and well-being: a cross-sectional observational study
Source: Eur Arch Otorhinolaryngol. 2024 Feb 23;281(7):3639–47. doi: 10.1007/s00405-024-08529-9 (PMC11211102; doi:10.1007/s00405-024-08529-9)
Supplement: Supplementary file 1 — Supplementary file1 (DOCX 1366 KB) [file 405_2024_8529_MOESM1_ESM.docx]

**Supplementary Table 1**

| Group | Food incidents | Gas incidents | Gas scares | Work scares |
| --- | --- | --- | --- | --- |
| NA | 159 | 72 | 72 | 72 |
| 0 | 134 | 296 | 211 | 280 |
| 1 | 36 | 41 | 72 | 32 |
| 2 | 36 | 13 | 41 | 24 |
| 3 | 18 | 5 | 13 | 8 |
| 4 | 11 | 2 | 6 | 1 |
| 5+ | 38 | 3 | 17 | 15 |

Table 1. Scares and incidents in the last 5 years numbers and percentage for main Figure 2.

**Supplementary Figure 1**


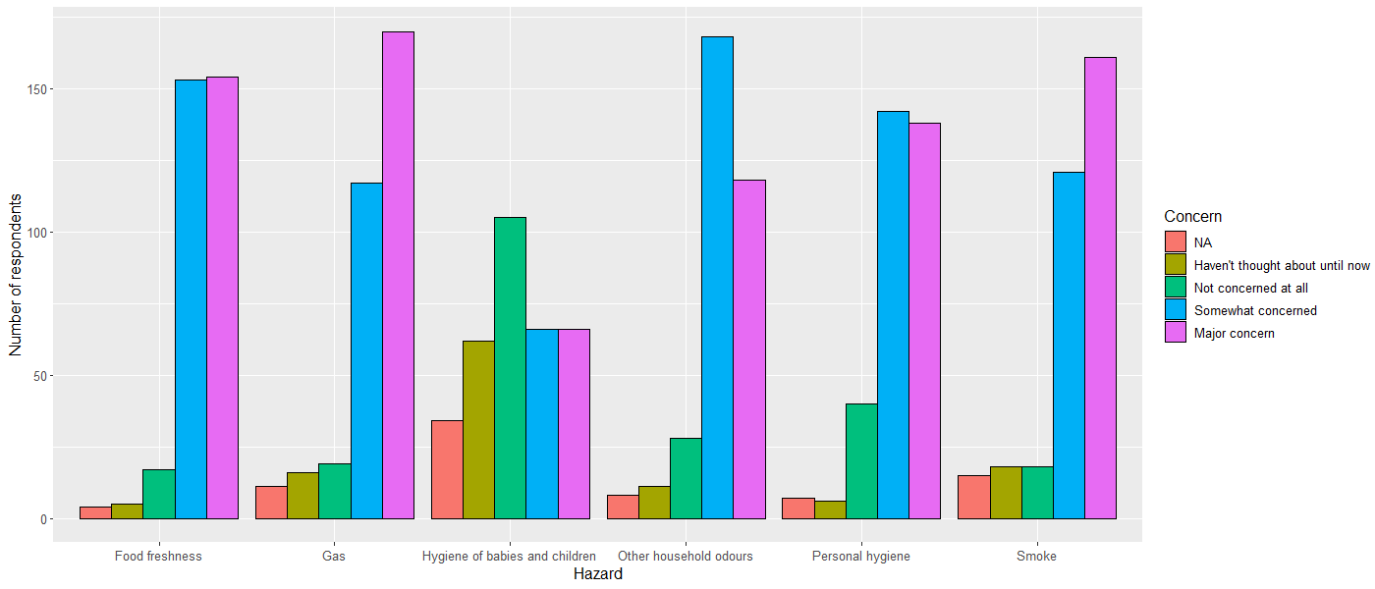


Figure 1. UK-subset figure for degree of safety concerns for gas, smoke, food freshness, personal hygiene, hygiene of babies and children, and other household odours (e.g. waste bins or pets). Numbers provided in table below.

**Supplementary Figure 2**


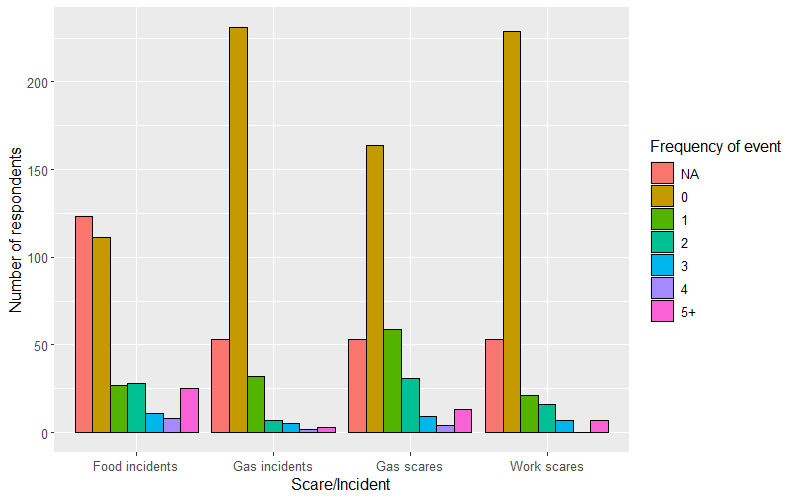


Figure 2. UK-subset figure for scares and incidents in the last 5 years. Numbers provided in table below.
